# Meeting Review: The Bioinformatics Open Source Conference 2001 (BOSC 2001)

**DOI:** 10.1002/cfg.107

**Published:** 2001-10

**Authors:** K. Cara Woodwark

**Affiliations:** Biomolecular Sciences UMIST PO Box 88 Manchester M60 1QD UK


**Comparative and Functional Genomics**
*Comp Funct Genom* 2001; **2**: 327–329.
**DOI:** 10.1002/cfg.107

**Feature**

### Meeting Review: The Bioinformatics Open Source Conference 2001 (BOSC 2001)

K. Cara Woodwark*
*Biomolecular Sciences, UMIST, Manchester, M60 1QD, UK*

*Correspondence to:
K.Cara Woodwark, Biomolecular Sciences, UMIST, PO Box 88, Manchester, M60 1QD, UK.
E-mail: kcw@bms.umist.ac.uk*

**Keywords:** bioinformatics; open source; Bioperl; BioJava; BioPython; CORBA

The general aim of the BOSC was to bring people up to date with what is happening in the open source community, which has representatives all over the world. Some of the talks were only five minutes long (Lightning Talks). These were a great idea as they gave many more people the opportunity to introduce a new area or update on an old one. However, their short length did mean that a certain level of knowledge was presumed.

BioPerl had perhaps the biggest presence but as the oldest Bio* (BioStar) that is not really surprising, although BioPython was close on its heels, with applications in many areas. Much of the open source collaborations are supported by the Bio* project using hardware provided by Sun and hosted by the Genetics Institute in Boston, USA. A general cry for more people to be involved with the coding seemed to be a common lament of all the Bio* projects.

All in all an enjoyable if rather quick (only two half days) and intense look at what's happening in the BOS Community. More details as well as abstracts of the talks and posters are available from **http://www.open-bio.org/bosc2001/.**

**Steve Brenner** from UCA Berkley started the conference with a talk on *The Open Source Author's Contract*. This was aimed at the Americans in the audience with unfortunately, no feed back from the different European countries as to what the equivalent situation is here, although it definitely gave us all food for thought. He pointed out that creating open source software at an American university is usually illegal, as the university often owns the copyright! Something not many of us had realised. Apparently, this is similar to the situation in many British universities.

He also put forward that there should be a standardised contract for coders to use that superseded university policy and which should be signed as a condition of employment, with the legal fees paid for by the open Bio consortium. This would be the responsibility of the group leader who could opt for open source, commercial or both so long as any sponsors agreed.

**Thomas Down** (Sanger Centre), introduced us to *BioJava*, a relative newcomer (in comparison to BioPerl) although there are already a large number of libraries available, including some for BLAST parsing (**http://org.BioJava.utils.query**).

**Andrew Dalke** (Dalke Scientific Software) gave an update on *BioPython: Status and Plans*. BioPython looks very interesting and seems to be quite an intuitive programming language. Python is a high level language like Perl, it is runtime, readable and easy to use. There are BLAST parsing capabilities; an SQL interface to BioPerl DB, and may be used for search and retrieval from e.g. GenBank (**http://www.biopython.org/**).

**Jason Stajich** (Duke University) presented a talk on *BioCORBA*. CORBA (Common Object Request Broker Architecture) is the industry standard to connect (wrap) different types of tools and databases, irrespective of what language they are written in. BioCORBA is the Bio* implementation of this. Recently BioCORBA wrappers have been written for EnsEMBL and EMBOSS. A BioPerl binder has been written to make BioCORBA Objects into BioPerl or BioJava objects and *vice*

Copyright © 2001 John Wiley & Sons, Ltd.

*versa*, which should prove very useful (**http://www.corba.org**).

The rest of the morning was given over to **Lightning Talks**. These were a series of 5-minute talks to introduce a new area or to update on an old one. **Catherine Letondal** (Pasteur Institute Computing Center) gave the first of the Lightning talks on *BioK*, which looked very interesting, as in theory its introspective capabilities actually allow the biologist to code by dragging and dropping components (objects) to make 'code' all within a GUI (graphical user interface). BioK is implemented in XOTcl – an object extension of Tcl (a set of Perl graphics modules). It is also compatible with Tk (another set of graphics modules) and uses its plot widgets. TkTable forms the basis for holding and viewing the data. Catherine showed us a table (from a multiple sequence alignment) of sequences coloured for promoter regions that a student had written after she had only been using BioK for one month. Unfortunately, BioK is only available at the Pasteur institute, at the moment, as it is still only a prototype but more details are available from **http://www-alt.pasteur.fr/~letondal/biok/**.

**David Block** (National Research Council Plant Biotechnology Institute) introduced us to *Genquire: live database-driven graphical objects*, which sounded very interesting, as it is OOPerl based and in theory the code writes its own code, by pulling attributes out of a database. For example, the table name becomes the name of the object. It even produces SQL queries on the fly and has an inherited dynamic SQL generator for loading tables with objects. However, the inheritance is very complex so there are still many bugs, but this is one to look out for in the future. Its progress can be followed at **http://bioinfo.pbi.nrc.ca/dblock/wiki.**

There were also Lightning Talks given by: **Scott Markel**, (NetGenics Inc) on *OMG's New Model Driven Architecture and Its Implications for the Life Sciences* Community (**http://doc.omg.org**); **Yoshinori Okuji** (Kyoto University) on *BioRuby* (**http://bioruby.org**), and **Iddo Friedberg** (Hebrew University of Jerusalem) on *Generation and Use of Substitution Matrices in Biopython.*

**Hilmar Lapp** (Genomics Institute of the Novartis Research Foundation, San Diego) started the second day of the conference with a talk entitled *BioPerl enters maturity and the post-genomics era.* The BioPerl project is the oldest of the open source Bio* projects having been started in 1995. Hilmar Lapp invited us all to join the BioPerl mailing lists and to get involved. The BioPerl policy (one now adopted by much of the bioinformatics open source community) is that 'he who codes it, wins the argument'. Which seems to have been a very satisfactory way of solving many design conflict problems. Jason Stajich is to create and maintain a script repository, along with packages that are built on top of BioPerl. BioPerl may be obtained from **http://www.bioperl.org/,** which also contains news and updates. A full stable release 1.0 will be out early in the last quarter of this year.

**Arne Stabenau** (EBI) presented *EnsEMBL: An open source project for genome annotation* on behalf of the EnsEMBL team. The EnsEMBL team are now involved with mouse genome annotation as well as human. The human genome is presently at 92% coverage. EnsEMBL is written mostly in Perl with a MySQL database (db) which produces downloadable flatfiles, which combined with a schema, available online, allow the recreation of the MySQL db, anywhere. The MySQL db is accessed via a PerlDBI access layer using adaptors (db access objects). However, they are hoping to do a Java port in the near future as this will allow: better OO support; compile time checking; multi-threading; easier maintenance; a better graphics library and hence better viewers; increased speed (at present their Java version is at least as fast as Perl), and an opportunity to clean up and streamline what has been a very organic system.

**Ewan Birney** (EBI) gave the first of the morning's Lightning Talks on *BioPerl db.* The BioPerl database (db) came about as a means of decreasing reliance on SRS for EnsEMBL. It is a relational db with sequences, features and annotation that are bound into BioPerl so that a BioPerl object becomes an instance in the db and vice versa. The next step is to try to completely recreate Embl or Genbank entries from the db. At present this is a beta release, which may change quite a bit before the first stable release.

**Martin Senger** (EBI) gave a lightning talk on *OpenBQS - Bibliographic Query Service (PII - Platform Independent Implementation*). Open BQS is the open source version of the Bibliographic Query Service based on the OMG (Object Management Group) standard, with a UML (unified modelling language) information model of bibliographical citations. Written in Java, it can be used with CORBA to access remote databases or without CORBA to access local tools in a Platform Independent Implementation (PII or Π). Tools are

Copyright © 2001 John Wiley & Sons, Ltd.

being developed to query Medline and the reference information from EMBL. UML: **http://industry.ebi.ac.uk/~senger/BQS/web** OBQS: **http://industry.ebi.ac.uk/openBQS.**

**Boris Lenhard** (Karolinska Institutet) introduced the *TFBS: Perl modules for transcription factor detection and analysis*. These Perl modules, which are completely compatible with BioPerl, can be used to create matrices to describe transcription factor binding sites, which can be used to search UTR's for protein binding sites. Creating matrices of conserved regions between a pair of homologues greatly increases specificity. The modules and further information are available from **http://forkhead.cgr.ki.se/cgi-bin/consite** and **http://forkhead.cgr.ki.se/TFBS.**

Other lightning talks were given by: **Juha Muilu** (EBI) on *OpenBSA - Tools and standards for distributed computing* (**http://www.omg.org/cgi-bin/doc?dtc/00-11-01**); **Peter van Heusden** (Electric Genetics) on *a tool for mining dbEST annotation using a controlled hierarchical vocabulary*, so ESTs can be "clustered" by their annotation, and by **Brad Chapman** (University of Georgia) on *Genetic Algorithms and Neural Network Libraries* (**http://www.bioinformatics.org/bradstuff**).

**Chris Mungall** (BDGP) described *A Tool Suite for the Gene Ontology* (GO). One of the many uses of ontologies (in this case gene ontologies) is to be able to make (biological) questions computable. The gene ontology is a relational database of 7000 terms (including synonyms) based on 3 main hierarchies – function, processes and cell locations. However as many genes are involved in many different processes and can at times be in different cellular locations, there is a certain amount of overlap, producing directed acyclical graphs. A graph is a linked collection of nodes and in this case the nodes are terms. Acyclical graphs are used as every node/term may have more than one parent, for example a protein may be described as an oxidoreductase and a transferase e.g. alcohol dehydrogenase (ADH, an enzyme that was much in demand at BOSC and ISMB2001).

The database is available in flatfile or mySQL/PostGres format and should be compatible with Oracle (untested). There is a Perl API (Application Programming Interface) for querying as well as java modules and a java interface to the editor. There is also a C version which is much faster than Perl but this is only available for the flatfile version, although they are currently working on a Perl binding between the C code and the relational db. The GO browsers are written in PerlCGI (or Python but again just flatfile) and they are working on/thinking about integrating it with EnsEMBL. More information is available from **http://www.fruitfly.org/annot/go/** (not *Drosophila* specific), **http://www.geneontology.org** and **http://www.godatabase.org.**

**Debra Goldberg** (Cornell University) introduced *DeCAL: An Open Source System for Constructing Comparative Maps*. DeCal is a method of comparing 2 genomes by sliding a window along each genome looking for homology. When a region of homology is encountered this forms the start of a homology block. The window then moves on and the question is asked is this window also homologous and if so is it homologous to the same region as the previous window thus increasing the size of the syntenous block. Or is it homologous to a new region – perhaps forming a new syntenous block, to a region on another chromosome, due to a translocation. The same question is asked in turn of each window to see whether to extend regions of synteny, start new blocks, allow for a small region of insertion or ignore as non-homologous. The algorithm is involved in answering these questions and has a colourful GUI (**http://www.cam.cornell.edu/~debra**).

---

The Meeting Reviews of *Comparative and Functional Genomics* aim to present a commentary on the topical issues in genomics studies presented at a conference. The Meeting Reviews are invited; they represent personal critical analyses of the current reports and aim at providing implications for future genomics studies.

Copyright © 2001 John Wiley & Sons, Ltd.

